# Important Medical Legislation

**Published:** 1890-08

**Authors:** 


### IMPORTANT MEDICAL LEGISLATION.

The *Journal of the American Medical Association*, in its issue of July 26, 1890, in a leader under the above title, ably discourses on the New Medical Examiners' Law as follows :

"The Medical Society of the State of New York is to be congratulated upon the success which, after seven years of heroic effort, it has achieved in the advancement of medical education. To this organization, more than to all other agencies or influences, the profession is indebted for the results that have been secured. In view of the action which has been taken by the House of Representatives, it did seem, for a time, as though in the Empire State, where least of all we should anticipate defeat, the hands were to be turned back upon the dial of medical progress, and that in the long-drawn struggle, illiteracy, joined hand-in-hand with selfish greed, was yet again to triumph. But, at last, the better counsels have prevailed, and congratulations are in order.

"The opposition to the present law, we regret to say, was not confined to sectarian schools alone, nor to such influences as charlatans would wield — but we think, unfortunately for them, some of our medical educators, who should have been conspicuous in their efforts to secure the passage of the bill, are numbered with the opposition ; seemingly from the fact that as the result of its beneficent working the patronage of their schools would be diminished — as it surely should and will be.

"The law approved by the Governor on June 4, 1890, will mark a new era not only in New York, but, incidentally, in the entire country. It cannot be said that in this contest, where every point has been fought out inch by inch, there is evidence of hasty and inconsiderate legislation. The utterances of this bill are like decisions of the Supreme Court, from which there should be no appeal.

"In the next number of the *Journal* we propose to present the bill entire, and we commend it to the careful consideration of our readers. As the result of such consideration, the first cardinal point which occurs to us is this: that the State presumes to reaffirm and to emphasize the fact that it alone has jurisdiction over the practice of medicine within its own limits. It may not only determine as to who may practise medicine, but it also presumes to assert what the qualifications of its practitioners shall be. In the exercise of this power it recognizes the responsibilities which it assumes, and seeks to make the best provision. In the second place, it proposes to redeem the profession from illiteracy by the requirement of a satisfactory preliminary education as a condition to an entrance upon the study of medicine. In the third place, it provides for a definite and uniform standard of examinations, and each and every student must attain to that standard as a condition to graduation. In the fourth place, it divorces medical teaching from the licensing power. To this end the examining boards are under the supervision of special examiners, who are appointed by the regents, and who themselves cannot be members of those boards. And, finally, it compels the colleges to teach their students three years, instead of two.

"We have, then, in this single bill, the requirements of preliminary education; a definite standard for examinations; a separation of teaching from the licensing power; and a three years' college course. Thus grandly has the State redeemed itself from what had threatened her, as a sad misfortune. It may be objected that among the provisions of this bill there has been an unwise recognition of sectarian schools. A careful study of the law gives evidence that there is one common power behind the separate boards, and that while they officiate as agents the power of decision rests alone in the Board of Regents. If this board shall perform its duties wisely and well, as we sincerely hope and anticipate, the State of New York will have little cause to fear from the illegitimate practice of medicine so long as the law shall remain in force. Besides, we are confident that a death-blow to sectarian medicine is never so wisely dealt, nor so sure of fatal results, as when by law all medical students must attain to a high standard of education, and by that standard *stand* or *fall*."

These are encouraging words, for which we beg to thank our contemporary, and we hope they will be carefully read and pondered by the profession throughout the State.

